# Nonlinear Dynamic Analysis of an Electrostatically Actuated Clamped–Clamped Beam and Excited at the Primary and Secondary Resonances

**DOI:** 10.3390/mi14101972

**Published:** 2023-10-23

**Authors:** Ayman M. Alneamy

**Affiliations:** Department of Mechanical Engineering, Jazan University, Jazan 45142, Saudi Arabia; alneamy@jazanu.edu.sa

**Keywords:** electrostatically actuator, subharmonic, period-doubling bifurcations, activation level

## Abstract

This work investigates the primary and secondary resonances of an electrostatically excited double-clamped microbeam and its feasibility to be used for sensing applications. The sensor design can be excited directly in the vicinity of the primary and secondary resonances. This excitation mechanism would portray certain nonlinear phenomena and it would certainly lead in increasing the sensitivity of the device. To achieve this, a nonlinear beam model describing transverse deflection based on the Euler–Bernoulli beam theory was utilized. Then, a reduced-order model (ROM) considering all geometric and electrical nonlinearities was derived. Three different techniques involving time domain, fast Fourier transforms (FFTs), and frequency domain (FRCs) were used to examine the appearance of subharmonic resonance of order of one-half under various excitation waveforms. The results show that higher forcing levels and lower damping are required to activate this resonance. We note that as the forcing increases, the size of the instability region grows fast and the size of the unstable region increases rapidly. This, in fact, is an ideal place for designing bifurcation inertia MEMS sensors.

## 1. Introduction

The investigation of primary and secondary resonances in electrostatic MEMS (microelectro-mechanical systems) actuators is indeed crucial for optimizing their performance [1]. Electrostatic MEMS actuators are devices that utilize electrostatic forces to generate mechanical motion at the microscale. They can be used in various applications, including filter [2], aerospace [3], biomedical [4], communication [5], and logic-gates [6].

One of the important phenomenons in MEMS is resonance, as it determines their operational characteristics, such as actuation frequency, power consumption, and overall efficiency [7]. There are two types of resonances appearing in MEMS, primary and secondary resonances [8]. It is known that primary resonances occur when the excitation frequency is close to one of the fundamental frequencies of the microstructure [9]. These resonances are of particular interest because they can significantly enhance the response of the MEMS actuator [10].

On the other hand, secondary resonance refers to a phenomenon in which a system exhibits resonance at a frequency that is half or twice the excitation frequency [11,12]. By utilizing a secondary resonance, it is possible to enhance the oscillation amplitude of the system. This technique has been employed to improve the performance of various devices, such as MEMS-based resonators and sensors [13].

However, when dealing with highly nonlinear electromechanical coupling, there are potential challenges that need to be considered. One such challenge is the increased susceptibility to pull-in instability. This occurs when the electrostatic force acting on a microstructure becomes so strong that it overcomes the mechanical restoring force and causes the structure to collapse or stick to the opposing surface. In addition, the secondary resonance has been used to improve the oscillation amplitude. However, due to the highly nonlinear electromechanical coupling and the growing demand of larger travel range for higher performance, there is a potential challenge that the device is more susceptible to pull-in instability [14].

It is known that, at large displacements, the electrostatic force can exhibit nonlinear behavior, resulting in deviations from linear actuation responses. This is commonly observed in devices such as electrostatically actuated microcantilevers, membranes, or comb-drive. Alternatively, nonlinear behavior can also arise from the geometric effects of microstructures. For example, when a resonator undergoes large vibrations, the deflection of the structure can cause changes in its stiffness, resulting in nonlinear frequency response with softening or hardening behavior [15].

The study of the two resonances appearing in electrostatic MEMS sensors involves a combination of theoretical analysis and experimental characterization where many researchers have employed various techniques [16,17]. For instance, analytical models taking into account the geometrical nonlinearities and electrostatic force have been developed to describe the system dynamics and to predict the resonance frequencies [18,19,20,21].

The work carried out by Younis and Nayfeh [22] focused on the analytical investigation of the primary resonance of an electrostatic microactuator. They studied the behavior of the actuator when excited at frequencies close to its fundamental resonance frequency. The theoretical findings suggested that exciting the electrostatic actuator near its superharmonic or subharmonic resonances could result in dynamic responses of similar magnitudes as primary resonance excitation. This implies that secondary resonances can be utilized to enhance the performance of MEMS actuators, providing alternative operational frequencies with substantial response amplitudes as well as highly sensitive devices.

Najar et al [23] also investigated the dynamic response of a microbeam electrostatic actuator. They employed a discretization technique that combines the differential quadrature method (DQM) and finite difference method (FDM) to analyze the actuator’s behavior. Through the combined DQM–FDM technique, they were able to generate frequency–response curves that captured the dynamic behavior of the microactuator under large excitations and over a wide frequency range. This allowed them to analyze the effects of nonlinearities, such as hardening or softening effect, on the actuator’s response.

The effectiveness of the nonlinear elastic and the inertia on the primary and secondary resonances of an electrostatically actuated clamped–clamped microbeam has been investigated analytically [24]. In this model, higher-order nonlinear terms are taken into account to provide insights about the dynamic motion that may not be captured by a linear approximation.

The research conducted by Kacem et al. [25] revolved around a dynamic stabilization technique for electrostatically actuated nanoresonators. The aim of their work was to stabilize the nanoresonators by simultaneously actuating both primary and superharmonic resonances. On the other hand, Taheri-Tehrani et al. [26] presented an interesting finding regarding mutual 3:1 subharmonic synchronization in a micromachined silicon disk resonator. This phenomenon refers to the synchronization of two coupled oscillators, where one oscillator oscillates at three times the frequency of the other.

Nowadays, the need to detect small objects has led many researchers to design new MEMS platforms. The sensitivity of these devices plays an important role and can be achieved using different techniques [27,28]. Here, we investigate an MEMS sensor excited electrostatically and near to the subharmonic resonance. To activate this resonance, higher force and lower damping effect are needed. The study involves an analytical investigation of a nonlinear equation of motion describing the transverse deflection of the clamped–clamped microbeam excited electrostatically. The analysis involves studying the effectiveness of subharmonic resonance and the influence of the excitation force on the dynamic response of the sensor. This has an impact on designing highly sensitive MEMS devices compared to those operated using traditional mechanisms. The results are presented in the time domain, fast Fourier transforms (FFTs), and frequency–response curves (FRCs).

## 2. Mathematical Model

**Figure 1:**
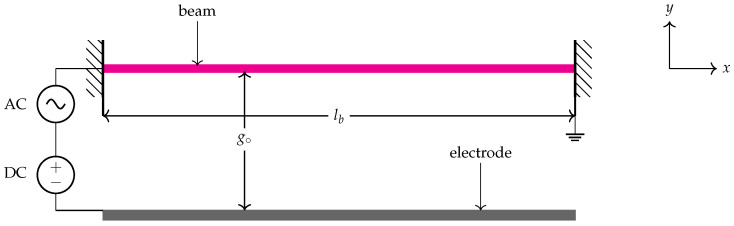
A schematic of the electrostatically actuated clamped-clamped microbeam.

The sensor consists of a clamped–clamped beam that is excited electrostatically via a side-wall electrode. Figure 1 shows a schematic for the its component. The sensor is made of single-crystal silicon. The straight microbeam has a length of $l_{b}=1000\;\mu m$, a width of b=30μm, and a thickness of h=3μm, while the capacitor gap between the sidewall electrode to the straight beam is set to $g_{\circ }=11.5\;\mu m$. The material Young’s modulus (*E*) is set to 129 GPa and its density (ρ) is set to 2332 kg/m${}^{3}$, respectively.

The microbeam is actuated by a direct electrostatic force that consists of a static voltage component DC, a time-varying voltage component AC, and an excitation frequency Ω sets at the primary, superharmonic of order-one-half and subharmonic of order-two resonances.

The equation of motion governing the transverse deflection of the beam midpoint ($\hat{w}$) is formulated in order to study the dynamic response of the sensor near the primary and secondary resonances. Following [29,30], the equation of motion can be written as

(1) $$\begin{array}{c} \begin{array}{cc} EI\frac{\partial^{4}\hat{w}}{\partial {\hat{x}}^{4}}+\rho A\frac{\partial^{2}\hat{w}}{\partial {\hat{t}}^{2}}+\hat{c}\frac{\partial \hat{w}}{\partial \hat{t}}= & \frac{\partial^{2}\hat{w}}{\partial {\hat{x}}^{2}}\left[\frac{EA}{2l_{b}}\int_{0}^{l_{b}}\left({\left(\frac{\partial \hat{w}}{\partial \hat{x}}\right)}^{2}+2\frac{\partial \hat{w}}{\partial \hat{x}}\right)d\hat{x}\right]+\frac{\varepsilon \;bV^{2}}{2{\left(d-\hat{w}\right)}^{2}} \end{array} \end{array}$$

The first three terms on the left-hand side of Equation (1) represent beam’s stiffness, mass, and viscus damping. The latter term can be expressed in terms of the damping ratio ζ. On the other hand, the two terms on the right-hand side represent the geometric nonlinearities and electrostatic force, respectively [8,31]. The area and moment of inertia of the cross-section are $A=bh_{b}$ and $I=bh_{b}^{3}/12$, respectively. *V* is the voltage waveform and can be expressed as

V=DC+ACcos(2πΩt)

The displacement and slope of the clamped–clamped beam at its end-supports are zero. These are the boundary conditions which are necessary for solving the boundary value problem

$$\hat{w}\left(0,\hat{t}\right)=0,\;\frac{\partial \hat{w}}{\partial \hat{x}}\left(0,\hat{t}\right)=0,\;\hat{w}\left(l_{b},\hat{t}\right)=0,\;\frac{\partial \hat{w}}{\partial \hat{x}}\left(l_{b},\hat{t}\right)=0$$

Then, the variables shown in the equation of motions $\left(\hat{x},\hat{t}\right)$ are normalized. As a matter of fact, the capacitor gap $g_{\circ }$ is a good candidate to normalize $\hat{x}$. On the other hand, the variable $\hat{t}$ is normalized with respect to the natural period of the system, defined by $T=\sqrt{\rho A{l_{b}}^{4}/EI}$. For convenience, we introduce the following nondimensional variables:

$$w=\frac{\hat{w}}{g_{\circ }},\;x=\frac{\hat{x}}{l_{b}},\;t=\frac{\hat{t}}{T}$$

After that, we substitute the nondimensional variables into the equation of motion to yield

(2) $$\begin{array}{ll} EI\frac{g_{\circ }}{l_{b}^{4}}\frac{\partial^{4}w}{\partial x^{4}}+\rho A\frac{g_{\circ }}{T^{2}}\frac{\partial^{2}w}{\partial t^{2}}+\hat{c}\frac{g_{\circ }}{T}\frac{\partial w}{\partial t} & =\frac{g_{\circ }}{l_{b}^{2}}\frac{\partial^{2}w}{\partial x^{2}}\left[\frac{EA}{2l_{b}}\int_{0}^{l_{b}}\left({\left(\frac{\partial w}{\partial x}\right)}^{2}+2\frac{\partial w}{\partial x}\right)l_{b}dx\right]\\ & +\frac{\varepsilon \;bV^{2}}{2\;g_{\circ }^{2}{\left(1-w\right)}^{2}} \end{array}$$

Thus, multiplying both sides of Equation (2) by ($T^{2}/\rho Ag_{\circ }$) results in

(3) $$\begin{array}{c} \begin{array}{c} \frac{\partial^{2}w}{\partial t^{2}}+c\frac{\partial w}{\partial t}+\frac{\partial^{4}w}{\partial x^{4}}=\alpha_{1}\frac{\partial^{2}w}{\partial x^{2}}\left[\int_{0}^{1}\left({\left(\frac{\partial w}{\partial x}\right)}^{2}+2\frac{\partial w}{\partial x}\right)dx\right]+\alpha_{2}F_{es} \end{array} \end{array}$$

where the nondimensional coefficients are

$$\alpha_{1}=6{\left(g_{\circ }/h_{b}\right)}^{2},\;\alpha_{2}=\frac{\varepsilon b\;l_{b}^{4}}{2EId^{3}},\;c=\frac{\hat{c}\;l_{b}^{4}}{EIT},\;F_{es}=\frac{V{\left(t\right)}^{2}}{{\left(1-w\right)}^{2}}$$

and the nondimensional boundary conditions are

(4) $$w\left(0,t\right)=0,\;\frac{\partial w}{\partial x}\left(0,t\right)=0,\;w\left(1,t\right)=0,\;\frac{\partial w}{\partial x}\left(1,t\right)=0$$

Because closed-form solutions are rare for systems governed by nonlinear equations, approximate methods of solutions need to be utilized to solve Equation (3). Therefore, a reduced-order model based on a Galerkin approximation is utilized to simulate the static and dynamic behavior of the sensor [16]. This technique discretizes the equation of motion in terms of a finite number of degrees of freedom describing the amplitude of modal shapes.

Additionally, the solution fidelity depends on the type and number of mode shapes used in the Galerkin approximation. These mode shapes must satisfy the natural boundary conditions where, in this case, we chose to utilize the mode shapes of a straight beam. Therefore, the solution of Equation (3) is assumed as

(5) $$w\left(x,t\right)=\sum_{n=1}^{K}\psi_{n}\left(x\right)u_{n}\left(t\right)$$

where *K* is the number of modes retained in the discretization process, $\psi_{n}\left(x\right)$ are the trial functions that satisfy the boundary conditions, and $u_{n}\left(t\right)$ are the generalized coordinates. Then, we multiply both sides of Equation (3) by ${\left(1-w\right)}^{2}$ to reduce the computational time and to regularize the response near the singularity. After that, we substitute Equation (5) into the resulting equation. Then, multiplying it by the mode shape $\psi_{j}$ on both sides and integrating along the beam length from x=0 to x=1, we obtain the following differential equations in terms of modal coordinates:

(6) $$\begin{array}{l} \int_{0}^{1}\left[\psi_{j}\sum_{n=1}^{N}\left(\psi_{n}{\ddot{u}}_{n}+c\psi_{n}{\dot{u}}_{n}+\psi_{n}^{iv}u_{n}\right){\left(1-\sum_{n=1}^{N}\psi_{n}u_{n}\right)}^{2}\right]dx=\\ \int_{0}^{1}\left[\alpha_{1}\psi_{j}\left(\sum_{n=1}^{N}\psi_{n}^{{}^{\prime \prime }}u_{n}\right)\times \int_{0}^{1}\left[{\left(\sum_{n=1}^{N}\psi_{n}^{{}^{\prime }}u_{n}\right)}^{2}+2\sum_{n=1}^{N}\psi_{n}^{{}^{\prime }}u_{n}\right]dx\right]dx+\alpha_{2}V^{2}\int_{0}^{1}\psi_{j}\;dx \end{array}$$

## 3. Results and Discussion

First of all, we carried out a static convergence analysis to compare the beam’s response obtained from ROMs, Equation (6), employing one-, two-, and three-symmetrical-mode configurations. This is a significant step in order to determine the minimal number of modes required in the Galerkin expansion.

**Figure 2:**
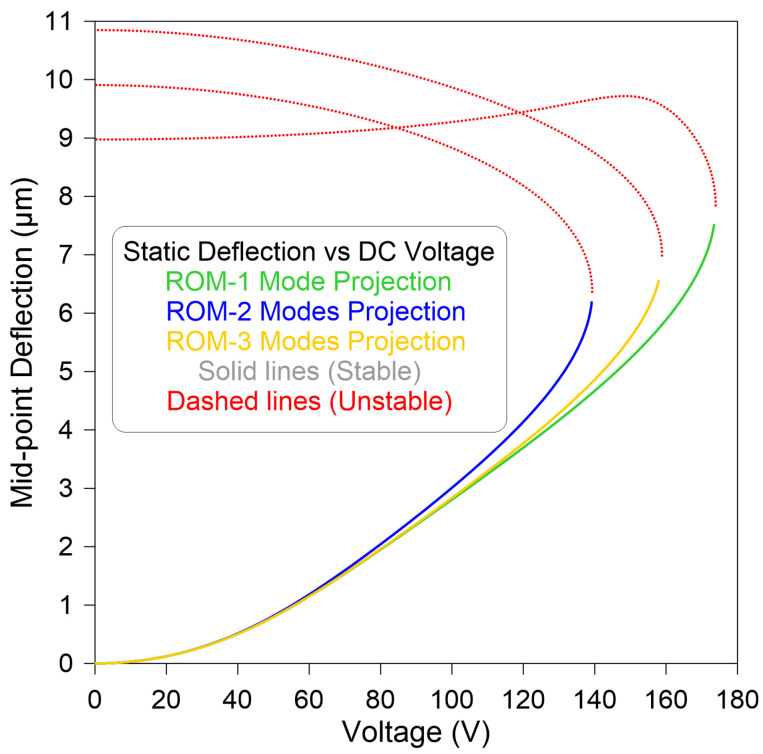
Convergence analysis of the ROM on the static deflection of the straight beam midpoint as a function of DC voltage, employing one symmetric mode (—), two symmetric modes (—), and three symmetric modes (—).

Figure 2 shows the variation in the midpoint deflection as a function of the static voltage DC for three developed ROMs. In all cases, only one branch of stable equilibria, marked as solid lines, and one branch of unstable equilibria, marked as dashed lines, were observed. The static results show that at least three symmetric modes, marked as yellow line (—), are required for satisfactory model convergence compared to lower-mode approximation, which results in quantitative error. This error is clearly visible along the unstable branch of equilibria.

Furthermore, to study the dynamic behavior of the sensor, we have to first compute the resonance frequencies of the sensor. This is achieved by numerically solving the free eigenvalue problem of the clamped–clamped beam using Mathematica software [32]. We found that the first in-plane bending resonance frequency of the sensor has a frequency of $f_{n}=22.93$ kHz.

Furthermore, to evaluate the dynamic response and to examine its stability, Equation (6) is numerically integrated for a long time until reaching steady-state response. In this case study, the actuated voltage corresponds to a single equilibrium point. A similar observation to that discovered statically was obtained. Exciting the sensor near the primary resonance of the first in-plane bending mode, it means that we are likely interested in understanding the dynamic behavior of the system at this specific mode. It is known that the primary resonance is of particular interest because it represents the most significant response of the system. This confirms that exciting the sensor at half of this value yields to superharmonic resonance and twice this frequency results in subharmonic resonance, respectively. They can be achieved using the electrostatic force which, in fact, is proportional to the voltage square. Two components exist: a lower harmonic component at $f_{n}$ with an amplitude of (∝1/2), and a higher harmonic component at $2f_{n}$ with an amplitude of (∝1/4).

The sensor is then excited using a lower-voltage waveform equal to DC = AC = 5 V and an excitation frequency near the primary and secondary resonances. The FFT signals of the numerically predicted velocity and corresponding to this excitation voltage were obtained numerically by integrating the equation of motion utilizing the long-time integration (LTI) technique with three-mode projection. This is performed over 2000 time periods. Then, the time history of the last 100 signal periods is recorded to obtain the steady-state response.

**Figure 3:**
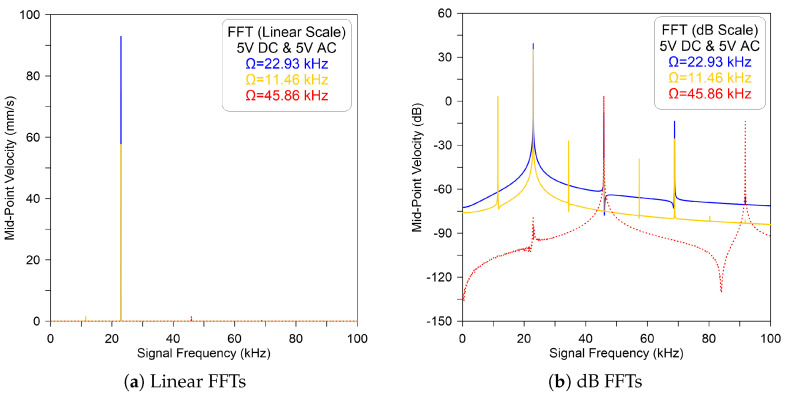
The model-predicted FFT velocity of the sensor excited by DC = AC = 5 V and excitation frequency of Ω=22.93 kHz (—), Ω=11.46 kHz (—) and Ω=45.86 kHz (- - -) recorded in (**a**) linear and (**b**) dB scales.

Figure 3a,b show the FFT signals in linear and dB scales, respectively, for the sensor’s velocity when the excitation frequency is set to Ω=22.93 kHz. It demonstrates evidence of the primary resonance with a large peak that appears at Ω for the first case, marked as blue line (—), and smaller peaks appearing at nΩ, where n=1…4. On the other hand, observing superharmonic resonance involves exciting the system with a signal frequency that is half of the fundamental frequency. In this case, we kept the voltage similar to the previous case and set the excitation frequency to Ω=11.46 kHz.

Figure 3 demonstrates evidence of superharmonic resonance of order two, marked as a yellow line (—), with resonance peaks appearing at nΩ where $n=(\frac{i}{0.5}-1)+0.5$, i=2…5. We note that the maximum response of the sensor appears at the resonance frequency $\Omega =f_{n}$ even though the forcing frequency is half of the excitation frequency. This, in fact, is a basic characteristic of superharmonic resonance.

In addition, increasing the signal frequency to Ω=45.86 kHz leads to a similar behavior with a large peak appearing at 2Ω, denoted by a dashed red line (- - -). This is an indicator that subharmonic resonance is not activated. It can be confirmed from the FFT signals, as shown in Figure 3a, for the linear scale and (b) for the dB scale, where only peaks appear at nΩ, where n=2i, i=1…2, and small peaks at half Ω. We note that the large harmonic peaks are clearly visible on the dB scale. Indeed, to activate subharmonic resonance, a higher forcing level is required.

**Figure 4:**
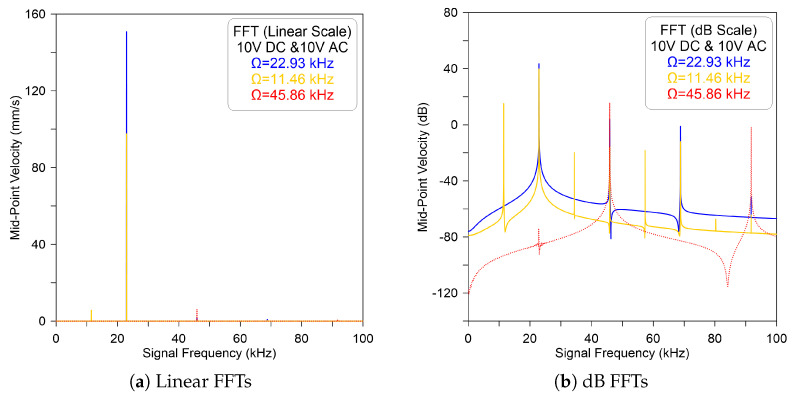
The model-predicted FFT velocity of the sensor excited by DC = AC = 10 V and excitation frequency of Ω=22.93 kHz (—), Ω=11.46 kHz (—), and Ω=45.86 kHz (- - -) recorded in (**a**) linear and (**b**) dB scales.

**Figure 5:**
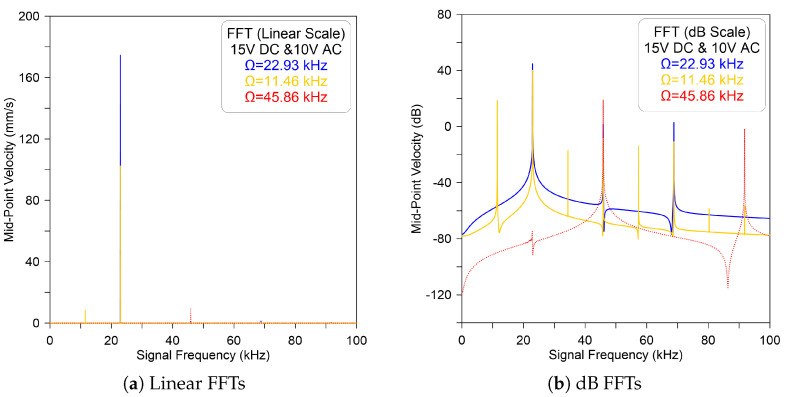
The model-predicted FFT velocity of the sensor excited by DC = 15 V, AC = 10 V and excitation frequency of Ω=22.93 kHz (—), Ω=11.46 kHz (—), and Ω=45.86 kHz (- - -) recorded in (**a**) linear and (**b**) dB scales.

A further increase in the excitation voltage, DC = AC = 10 V, leads to a similar dynamic response. However, a significant amplitude magnification was observed at the primary and secondary resonances. This, in fact, is expected because the electrostatic force has a higher effect than the previous case, as clearly illustrated in Figure 4. On the other hand, exciting the microbeam with a voltage waveform of DC = 15 V, AC = 10 V, and a signal frequency near to the vicinity of primary and secondary harmonic resonances results in a similar response to that observed for the previous two cases, as shown in Figure 5.

**Figure 6:**
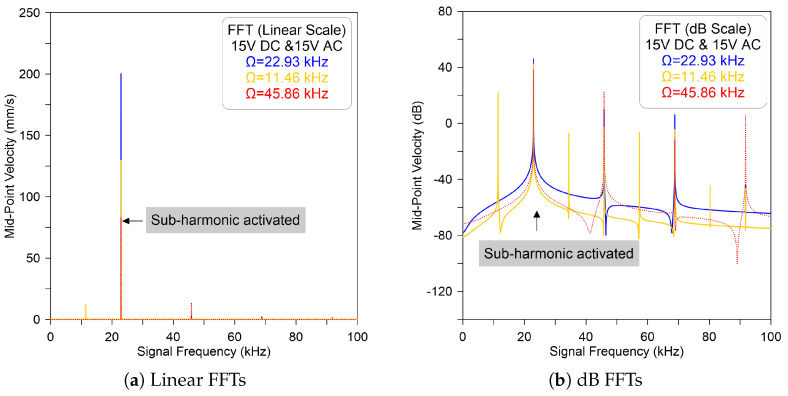
The model-predicted velocity in FFT signal of the sensor excited by DC = AC = 15 V and excitation frequency of Ω=22.93 kHz (—), Ω=11.46 kHz (—), and Ω=45.86 kHz (- - -) for (**a**) linear and (**b**) dB scales.

Alternatively, to examine the appearance of subharmonic resonance, the excitation force must exceed a threshold called activation level. Below this threshold, the subharmonic resonance is not activated and the branch of nonresonant responses is continuous and interrupted by instability. We note that under DC = AC = 15 V and a signal frequency of 2Ω, the FFT signals show an evidence of subharmonic resonance of order one-half, as shown in Figure 6a for the linear scale and (b) for the dB scale. It also shows that the ratio between the motion amplitudes at Ω and 2Ω are relatively large, as demarcated by a dashed red line (- - -). This nonlinear phenomena is a basic characteristic of the subharmonic resonance because the energy is transmitted from the excitation frequency 2Ω to the resonant frequency and begins to be localized at this region.

**Figure 7:**
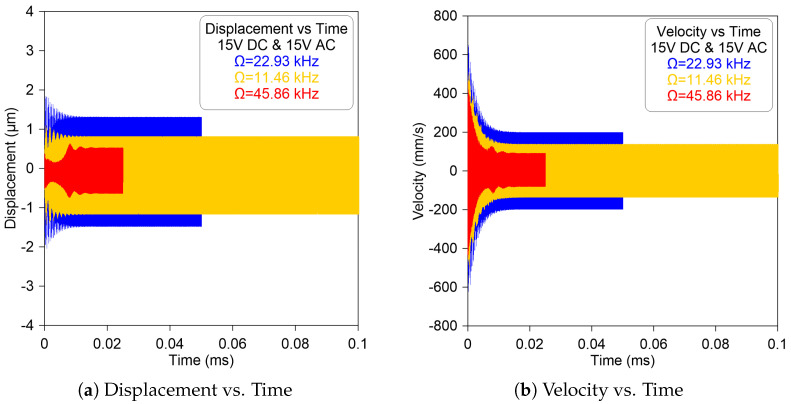
The model-predicted time-histories of the sensor: (**a**) displacement and (**b**) velocity excited by DC = AC = 15 V and signal frequency sets to Ω=22.93 kHz (—), Ω=11.46 kHz (—), and Ω=45.86 kHz (- - -).

In addition, the time histories at DC = AC = 15 V of the sensor’s midpoint displacement and velocity are shown in Figure 7a,b, respectively. The appearance of a sudden expansion in the displacement envelope is evidence that the lower harmonic component with a single period has activated the subharmonic resonance of order one-half, resulting in a resonant response with a period equal to twice the excitation frequency. The figure shows that the displacement and velocity are perfectly proportional to each other at any giving excitation frequency.

The dynamic response of the sensor was investigated by subjecting it to a frequency-sweep test in the vicinity of the subharmonic resonance of the first in-plane bending mode. Then, the variations in the amplitude of the sensor midpoint velocity under different excitation waveforms and a signal frequency in a range of 40–54 kHz were evaluated using the shooting method to generate periodic orbits and to determine their stability by evaluating their Floquet multipliers.

**Figure 8:**
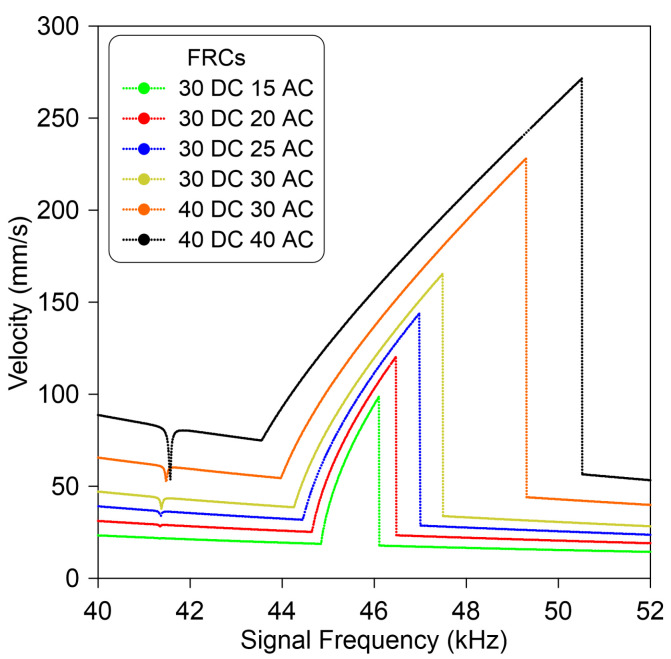
The model-predicted frequency–response curves of the double-clamped microbeam in the vicinity of the subharmonic resonance excited through different voltage waveforms.

For all voltage levels, Figure 8 shows that when sweeping down the frequency, the response increases along the lower branch of stable solutions until it hits a period-doubling bifurcation, corresponding to a Floquet multiplier that exists in the unit circle through −1. Then, it jumps to a higher branch of stable solutions and continues decreasing until it leaves the vicinity of subharmonic resonance. This phenomenon is observed at different voltages. However, as the forcing increases, the size of the unstable region increases rapidly. This, in fact, is an ideal site for designing inertia MEMS sensors, where any change in added mass results in a rapid jump from the lower stable branch solution to the upper stable branch solution through a bifurcation point.

## 4. Conclusions

This paper investigated the primary and secondary resonances of an electrostatically excited double-clamped microbeam and explored its potential for sensing applications. The sensor design aims to utilize excitation near the primary and secondary resonances, which would introduce nonlinear phenomena and increase the sensitivity of the device. The study employed a nonlinear beam model based on the Euler–Bernoulli beam theory to describe the transverse deflection of the microbeam. Additionally, a reduced-order model (ROM) considering all geometric and electrical nonlinearities was developed. To examine the appearance of secondary resonance, subharmonic of order one-half, time-domain analysis, fast Fourier transforms, and frequency–response curves were deployed. The results indicate that higher forcing levels and lower damping are required to activate this resonance. We found that the size of the instability region increased rapidly as the forcing level increased. This finding suggests that the device could be suitable for the design of bifurcation inertia MEMS sensors, which rely on the presence of unstable regions for their operation.
